# Post-Quantum Linkable Hash-Based Ring Signature Scheme for Off-Chain Payments in IoT

**DOI:** 10.3390/s25144484

**Published:** 2025-07-18

**Authors:** Linlin He, Xiayi Zhou, Dongqin Cai, Xiao Hu, Shuanggen Liu

**Affiliations:** 1 School of Software, East China University of Technology, Nanchang 330013, China; lilyhe@ecut.edu.cn; 2 School of Cyberspace Security, Xi’an University of Posts and Telecommunications, Xi’an 710121, China; zhouxiayi@stu.xupt.edu.cn (X.Z.); cai5102@stu.xupt.edu.cn (D.C.); huxiao@stu.xupt.edu.cn (X.H.)

**Keywords:** IoT, blockchain, post-quantum cryptography, off-chain payment, ring signature

## Abstract

Off-chain payments in the Internet of Things (IoT) enhance the efficiency and scalability of blockchain transactions. However, existing privacy mechanisms face challenges, such as the disclosure of payment channels and transaction traceability. Additionally, the rise of quantum computing threatens traditional public key cryptography, making the development of post-quantum secure methods for privacy protection essential. This paper proposes a post-quantum ring signature scheme based on hash functions that can be applied to off-chain payments, enhancing both anonymity and linkability. The scheme is designed to resist quantum attacks through the use of hash-based signatures and to prevent double spending via its linkable properties. Furthermore, the paper introduces an improved Hash Time-Locked Contract (HTLC) that incorporates a Signature of Knowledge (SOK) to conceal the payment path and strengthen privacy protection. Security analysis and experimental evaluations demonstrate that the system strikes a favorable balance between privacy, computational efficiency, and security. Notably, the efficiency benefits of basic signature verification are particularly evident, offering new insights into privacy protection for post-quantum secure blockchain.

## 1. Introduction

With the rapid development of blockchain technology, decentralized payment systems have gradually become an important research direction in the fields of finance and the Internet of Things (IoT) [1]. Blockchain has become a popular choice in the finance, supply chain, and intelligent transportation sectors due to its anti-counterfeiting and transparency features, as well as its ability to provide untrusted transaction records [2]. However, as the technology evolves, blockchain privacy and efficiency issues are increasingly coming to the fore, especially in off-chain payment systems, where off-chain payments (OCPs) can reduce the number of on-chain transactions, increase throughput, and reduce costs, making blockchain an important tool for scaling systems, but posing several challenges in terms of privacy and verifiability [3]. Traditional privacy protection methods based on public key cryptography, such as zero-knowledge proof (zk-SNARK) [4], have made some progress in off-chain payments. However, existing privacy protection mechanisms are usually plagued by problems such as transaction path disclosure and data tracking on the chain, which limits their effectiveness in protecting users’ privacy [5]. Ring signature technology is a noteworthy solution for further improving privacy protection. Although ring signatures can provide verifiable anonymity by hiding the signer within a group of signers, existing ring signature systems lack effective linkages, making it impossible to track and correlate a series of payment transactions. This means that it is not possible to determine whether two payments were initiated by the same signer, which can lead to duplicate payment problems, especially for large automated transactions in IoT scenarios [6]. In addition, existing cryptographic techniques face unprecedented security threats due to the continuous development of quantum computing technologies. In particular, public key cryptographic algorithms (such as RSA and ECC) rely on the computational complexity of integer factorization problems and discrete logarithms [7]. However, Shor’s algorithm (Peter Shor, 1994) has shown that quantum computers can solve these problems in polynomial time, completely outperforming conventional public key cryptographic methods [8]. This poses a serious threat to the blockchain, authentication, and digital signature systems that currently rely on public key cryptography: longer life cycles (e.g., smart meters, connected cars) are more vulnerable to future quantum attacks [9]. Therefore, there is an urgent need to research and implement cryptographic methods resistant to quantum computing attacks to ensure that blockchain and IoT payment systems remain secure in the future. NIST is currently conducting standardization work in the field of post-quantum cryptography, in which hash function-based signature schemes (e.g., SPHINCS+) are considered one of the most promising candidates for post-quantum signatures [10], and XMSS, as a sub-algorithm of SPHINCS+, is also considered a promising post-quantum signature scheme [11].

To address the above challenges, this paper proposes a linkable hash-based ring signature scheme tailored for off-chain payments in IoT environments, aiming to achieve both privacy preservation and efficient auditability of payment behaviors.

This work focuses on designing a post-quantum secure linkable ring signature scheme that is suitable for resource-constrained IoT devices. To detect double-spending while preserving user anonymity, we introduce a lightweight signature linking mechanism that allows the identification of repeated signers without revealing their identities. Through a comparative analysis with existing linkable ring signature (LRS) schemes, our scheme demonstrates significant advantages in terms of security and computational performance, making it well-suited for future blockchain payment systems that demand both efficiency and privacy protection.

### 1.1. Related Works

(1) Privacy-preserving mechanisms for off-chain payments

Off-chain payment solutions have been proposed to address blockchain scalability and transaction latency issues, aiming to enhance transaction efficiency and reduce on-chain congestion. In IoT environments, off-chain payments help reduce the frequency of on-chain transactions for smart devices, thereby lowering computational and storage overhead and improving transaction throughput. In intelligent transportation systems, for example, connected vehicles can process microtransactions, such as tolls or refills, via off-chain payments without having to send each transaction to the blockchain. In the Industrial Internet of Things (IIoT), devices in smart factories can handle payments for automated manufacturing services through off-chain channels, thereby improving overall system efficiency.

In this context, M. Green proposed BOLT, an anonymous payment channel [12], with a method that combines blind signatures and zero-knowledge proofs to realize anonymous payments. Later, G. Malavolta et al. studied the competition and privacy issues in payment channel networks (PCNs) [13] and proposed a protocol based on hash event blocks to improve privacy protection. However, this method does not support multi-hop payments and incurs high computational and storage overhead. To address the problem of multi-hop payments and interoperability between chains, reference [14] proposed Anonymous Multi-Hop Locking (AMHL) as an enhancement to HTLC that improves payment privacy but does not extend to multi-pass payments. The Lockable CryptoMaze protocol ([15]) and its improved version ([16]) aim to reduce computational overhead and ensure non-linkability between multiple payments by allowing senders to process their own data. However, computational efficiency still needs optimization, especially in IoT scenarios with resource-constrained devices. LightPay [17] was proposed to support multi-hop payments, achieving atomicity through adapter signatures based on the discrete logarithm problem, but it did not consider the impact of quantum computing attacks and future IoT payments in a quantum computing environment—hidden security threats. A post-quantum adapter signature based on isogenies for off-chain payments is proposed in [18]. However, it still suffers from high computational and storage costs, and its long-term post-quantum security remains under evaluation. In addition, reference [19] combines ring signatures with commitment schemes to enhance privacy, but introduces new challenges in traceability and system scalability. Reference [20] designed a secure ring signature scheme that uses Distributed Key Generation (DKG) and Elliptic Curve Cryptography (ECC) to reduce the risk of identity association and eliminate dependence on trusted third parties but needs to optimize computational scalability and revocation mechanisms to adapt to the requirements of large-scale payments in IoT scenarios.

Current schemes either struggle to effectively balance anonymity, post-quantum security, and resource constraints, or lack a practical deployment pathway. Moreover, ring signatures have not been systematically integrated into off-chain payment paths, with the absence of linkability mechanisms hindering behavior tracking.

(2) Linkability, traceability, and double-spending detection

Due to their anonymity and verifiability, ring signatures are widely employed to enhance privacy in blockchain systems. They also enable anonymous authentication and payment in IoT scenarios. For instance, in smart city environments, ring signatures can facilitate privacy-preserving IoT payments, allowing vehicles to pay for parking or refueling without disclosing identity information. In smart healthcare systems, ring signatures can safeguard access to medical data, ensuring both anonymity and traceability. Reference [21] proposed a lattice-based Linkable Ring Signature, L2RS-CS, which combines distributed key generation (DKG) and public key aggregation (PKA) techniques to improve signature security and compression efficiency. Reference [22] further improved linkable ring signature (LRS) to improve post-quantum security and rigorous security testing. However, there are challenges regarding computational overhead and practical implementation, which is especially important for IoT devices with limited resources. The authors of [23] proposed the DualRing ring signature method combined with SM2 signatures to optimize efficiency. This approach reduces the signature size from a linear to a logarithmic scale, improving efficiency and connectivity. It is suitable for anonymous transactions and electronic voting in blockchains. However, further improvements regarding quantum tolerance and computational effort are needed. In addition, an identity-based pluggable dual-loop signature (LDRS) was developed to address the dual voting problem encountered in dual payments and e-voting [24]. This method supports threshold signature extensions but still incurs high computational and communication overhead, making it unsuitable for low-power IoT devices. In addition, a STARK-based linkable ring signature (LRS) framework was proposed in [25], which optimizes signature size and computational efficiency and provides post-quantum security but with high computational complexity. Reference [26] proposes an efficient ring signature scheme that combines homology and lattice assumptions with log-OR testing to improve scalability and linkability but leaves room for further optimization, especially for large-scale inter-node communication applications in the IoT. A post-quantum blockchain framework that combines post-quantum PoW, storage mining and identity-based signatures and is applicable to smart cities and IoT, but without off-chain payments, was analyzed in [27]. Reference [28] proposed an efficient aggregated ring signature (ARS) and its application to secret transaction protocols (ARSCT) to improve transaction protection and verification efficiency. However, distributed aggregation schemes and quantum-resistant extensions have yet to be explored.

While most existing schemes provide anonymity, they lack the ability to detect or identify double spending. Linkable ring signatures have not yet been tightly integrated with HTLC or payment processes, resulting in a lack of practical, application-level deployment.

(3) Post-quantum signature scheme for blockchain and IoT

Regarding post-quantum blockchain signature schemes, the authors of [29] explored the application of schemes such as Falcon, Dilithium and XMSS to IoT and embedded systems, with Falcon optimized to reduce transaction sizes. However, these schemes still face challenges related to signing speed, state management, and zk-SNARK compatibility. Reference [30] proposed one-time hash-based signature chaining (OTS+ Naor-Yung Chaining) that optimizes the computational and storage overhead of the SPHINCS+ scheme to make it suitable for blockchain environments and resource-constrained systems. It has the potential to be applied to IoT devices, but it still faces problems with signature size, computational complexity, and selection of quantum-resistant hash functions. In [31], a traceable lattice-based ring signature scheme with superior signature size and computational efficiency was developed and applied to post-quantum blockchain access control. However, it has not been extended to the broader quantum-resistant security scenario. For example, hash signatures can ensure data integrity in smart sensor networks, while the Industrial Internet of Things (IIoT) can leverage them for device authentication to prevent the threat of quantum computing attacks on industrial control systems. Reference [32] looks in detail at the SPHINCS+ architecture, including the WOTS+, Hypertree and FORS components, and its application to post-quantum security. In [33], we propose Smart Digital Signatures (SDS), a compact and efficient hash-based signature scheme for Distributed Ledger Technology (DLT) that replaces ECDSA as a protection against quantum computing attacks. For the signature size optimization problem, SPHINCS-a and SPHINCS+C were proposed in [34,35] to optimize the signature size and improve computational efficiency. As part of the study of quantum signature algorithms after localization, reference [36] improves the XMSS algorithm with SM3 hash functions suitable for localization applications. However, challenges remain in signature size, computational complexity, and privacy enhancement. These issues may be mitigated through integration with zk-SNARKs in future work.

Most signature schemes fail to simultaneously meet the requirements of post-quantum security and lightweight deployment in IoT scenarios. Additionally, the signature structures lack tight coupling with payment processes such as HTLC and zero-knowledge proofs, making them unsuitable for anonymous payment applications.

(4) Although existing studies have made progress in enhancing privacy and post-quantum security in blockchain-based IoT payments, most solutions either lack quantum resistance or incur high computational costs, making them unsuitable for resource-constrained devices. Moreover, few schemes support efficient double-spending detection. This work fills that gap by integrating linkable ring signatures, hash-based post-quantum cryptography, and off-chain payment mechanisms for the first time. We design a double-spending detection algorithm compatible with HTLC, achieving strong anonymity, low overhead, and practical deployability, offering a new approach for building secure and auditable post-quantum payment systems.

### 1.2. Contributions and Innovations

This work presents the following key contributions and innovations:

First, we propose a post-quantum linkable hash-based ring signature scheme tailored for off-chain blockchain payment systems. The scheme achieves efficient anonymity and linkability while providing post-quantum security against potential quantum computing attacks.

Second, we design novel variants of Hash Time-Locked Contracts (HTLCs) enhanced by zk-SNARKs, which significantly improve the privacy of payment routing. These improvements enhance the anonymity of off-chain transactions and reduce exposure to traceability attacks.

Third, we conduct a comprehensive security and performance analysis of the proposed system. Through experimental evaluation in both blockchain and off-chain scenarios, we demonstrate that our scheme achieves notable advantages in verification efficiency compared to existing approaches.

This study further contributes the following original innovation:

To the best of our knowledge, this is the first work to integrate linkable ring signatures, post-quantum hash-based constructions, and off-chain payment mechanisms in a unified framework. We design a double-spending detection algorithm that is compatible with the HTLC protocol and supports efficient, anonymous, and auditable off-chain payments with practical deployment potential.

Moreover, we clarify the implications of our scheme for advancing research on post-quantum secure anonymous payment mechanisms, especially in resource-constrained IoT and blockchain environments.

### 1.3. Organization

The rest of the paper is organized as follows. Section 2 introduces the knowledge of linkable ring signatures, privacy protection in blockchains, and post-quantum signature algorithms. Section 3 proposes a hash-based post-quantum linkable ring signature scheme and explains its structure and security features in detail. Section 5 analyzes the proposed scheme from a security perspective and evaluates its resistance to quantum computation, forgery, anonymity, and linkability. Its suitability for practical use is examined. Finally, Section 7 summarizes the research results and examines possible future improvements.

## 2. Preliminary

### 2.1. Linkable Post-Quantum Ring Signatures

**Definition** **1.** *A ring signature is a digital signature technique that provides anonymity and allows a signer to generate signatures from a group (ring) without revealing his or her true identity. It was proposed by Rivest, Shamir, and Tauman in 2001 and is widely used for privacy preservation, such as anonymous transactions, e-voting, etc. [37].*

**Definition** **2.** *A linkable ring signature is an augmented ring signature that, in addition to providing anonymity and unforgeability, has the additional benefit of linkability. If the same user signs different messages, it is possible to somehow recognize that the signatures belong to the same user without exposing the specific identity of the user [38].*

The linkable ring signature procedure consists of four algorithms:

(1) Key generation (KeyGen): This algorithm generates public and private key pairs. Algorithm 1 ensures that the linkable tag lt is a hash of the secret key and that the same secret key generates the same lt with different signatures.
(2) Signature generation (Sign): Algorithm 2 generates a ring signature based on a specific key pair and a ring member with a linkability tag (LT).
(3) Verify signature (Verify): The procedure of Algorithm 3 can be used to verify whether the signature is valid.
(4) Linkability check: In this step, we determine whether two signatures are generated by the same private key. If the LT of the two signatures is the same, it can be determined that they come from the same private key, thus achieving linkability.

**Algorithm 1** Key generation (KeyGen).**Input:** None**Output:** sk, pk, lt1:sk←random_secret_key()         ▹ Generate the private key2:pk←public_key_from(sk)           ▹ Derive the public key3:lt←H(sk)              ▹ Calculate the linkability tag4:**return** (sk, pk, lt)
**Algorithm 2** Signature Generation (Sign)**Inputs:** m, sk, pk, R**Output:** (s, L, LT, R)  1:N=len(R)  2:h=H(M)           ▹ Calculates the hash value of the message  3:$y=[random_{value}\left(for_{in}range\left(N\right)\right)]$         ▹ Generate random values  4:s=[None]∗N  5:L=[None]∗N  6:LT=H(sk)                  ▹ Calculation of linkable labels  7:                    ▹ Computational ring structure  8:**for** *j* in range(N) **do**  9:     **if** R[j]==pk **then**10:          i=j                      ▹ Find the index11:          **continue**12:     $s\left[j\right]=random_{secretvalue}\left(\right)$13:     L[j]=H(y[j])+H(R[j])+s[j]14:     $s\left[i\right]=solve_{for_{si}}\left(h,L,sk\right)$               ▹ Calculation of $s_{i}$15:     L[i]=H(y[i])+H(pk)+s[i]16:**return** (s, L, LT, R)                ▹ Additional LT included
**Algorithm 3** Signature verification (verify).**Inputs:** M, s, L, LT, R**Output:** 0/11:h=H(M)2:$computed_{L}=\left[\right]$3:**for** *j* in range(len(R)) **do**4:     $computed_{L.append}\left(H\left(H\left(R\left[j\right]\right)+s\left[j\right]\right)\right)$5:**if** $H(computed_{L})==h$ **then**6:     **return** 17:**else**8:     **return** 0

### 2.2. Off-Chain Payments and Privacy Protection in Blockchain

Off-chain payments (OCPs) are a mechanism for conducting transactions outside the main blockchain to increase transaction efficiency, reduce fees, and improve privacy. They are typically used in payment channels and more complex payment networks (e.g., Lightning Network) to reduce dependence on the main blockchain and interact with it only when necessary.

Currently, off-chain payment solutions such as the Lightning Network have been adopted by several cryptocurrency exchanges and innovative Web3-oriented enterprises. For example, platforms like Kraken, Bitfinex, and Binance have integrated the Lightning Network into their deposit and withdrawal systems [39]; in 2021, Twitter incorporated Strike to enable Bitcoin tipping functionalities [40]; and the payment company Strike has collaborated with Visa and NCR to promote Lightning-based retail payments [41].

**Figure 1:**
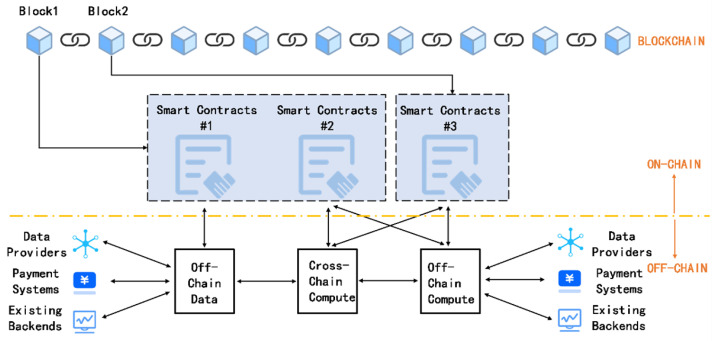
Schematic representation of blockchain and off-chain payments.

As shown in Figure 1, by minimizing on-chain transactions, off-chain payments improve transactions per second (TPS), reduce transaction fees by avoiding miner charges, and enable instant finality without block confirmation delays. Moreover, off-chain payment systems can be integrated with zk-SNARKs to enhance privacy and further reduce the need for on-chain interactions [42].

The post-quantum linkable hash-based ring signature scheme proposed in this paper for off-chain payments in the IoT holds significant implications for the broader economy. Featuring a lightweight design, quantum resistance, and privacy preservation, the scheme is well-suited for payments between resource-constrained IoT devices. Furthermore, it enhances the security and trustworthiness of off-chain payment networks. By providing a robust cryptographic foundation for future quantum-secure financial infrastructures, the proposed scheme supports the evolution toward next-generation payment systems that are both forward-compatible and regulation-friendly across various industries.

### 2.3. Signature of Knowledge

Signature of Knowledge (SoK) is a signed version of zero-knowledge proof (ZKP), which is an important tool in cryptography that not only verifies the authenticity of a message but also proves that “the signer actually possesses a certain secret message” and, on the contrary, the outside world cannot extract that secret message directly from the signature σ to extract that secret message directly [43].

**Definition** **3** (Knowledge Signature). *A knowledge signature consists of a triple (Gen, Sign, Verify), and these three algorithms are described below:*

*Parameter Generation $pp\leftarrow Gen(1^{\lambda })$:*

*In the parameter generation algorithm, the security parameter λ is the input, and the public parameter pp is the output.*

*Signature generation σ←Sign(pp,x,w,m):*

*In the signature generation algorithm, input the public parameter pp, the statement x, the witness w, and the message m, and then output the signature σ.*

*Signature Verification 0/1←Verify(pp,x,σ,m):*

*In the signature verification algorithm, the public parameters pp, statement x, signature σ, and message m are input, and a Boolean value (0 for rejection, 1 for acceptance) is output. Intuitively, if σ is a valid signature, the verification should pass with an output of 1. Otherwise, the output is 0.*

In general, a secure knowledge signature scheme must satisfy three fundamental properties: correctness, simulatability, and extractability.

**Definition** **4** (Correctness). *Correctness guarantees that if a signature is generated correctly according to the protocol, then it must pass verification, i.e., if a signature is generated by a legitimate algorithm, then it must be correctly verified and generated correctly according to the protocol, then it must pass verification, i.e., if a signature is generated by a legitimate algorithm, then it must be correctly verified:*

(1) $$\begin{array}{c} \mathrm{Pr}[Verify\left(pp,x,\sigma ,m\right)=1|pp\leftarrow \mathrm{Gen}\left(1^{\lambda }\right),\;\sigma \leftarrow \mathrm{Sign}\left(pp,x,\omega ,m\right)]=1 \end{array}$$

**Definition** **5** (Simulatability). *Simulatability requires that a knowledge signature can be “simulated” without actually knowing the witnesses. i.e., it is possible to define a simulator that generates signatures that appear to be “real” without relying on actual witnesses, i.e., a simulator can be defined that can generate “real”-looking signatures without relying on the authentic witness w:*

*The simulator first performs simulated parameter generation, inputting the security parameter λ and outputting the public parameter pp and a simulated trapdoor τ: $\left(pp,\tau \right)\leftarrow SimG\left(1^{\lambda }\right)$.*

*Then, the simulated signature algorithm, which inputs the public parameter pp, the simulated trap τ, the statement x, and the message m, and outputs the simulated signature σ: σ←SimS(pp,τ,x,m).*

*Any polynomial-time attacker A’s behavior is indistinguishable if he accesses the actual signer S and the simulator Sim. That is, it satisfies the following equation:*

(2) $$\begin{array}{c} \mathrm{Pr}\left[1\leftarrow A^{Sim(pp)}\left(pp,\tau \right)\right]\approx \mathrm{Pr}\left[1\leftarrow A^{Sim(pp)}\left(pp\right)\right] \end{array}$$

*This formula shows that even if an attacker had access to a simulator, he would not be able to distinguish between simulated and real signatures.*

**Definition** **6** (Simulated Extractability). *Simulated extractability ensures that if attacker A generates a valid signature, the signer “knows” w and can extract the witness w from this signature.*

To accomplish this, first define an Extractor Ex: w←Ex(pp,τ,x,m,σ), an algorithm that takes the public parameter pp, the simulated trapdoor τ, the statement *x*, the message *m*, and the signature σ supplied by the attacker as inputs, and outputs the witness *w*. This property requires that if the attacker A can successfully generate a signature σ, then the extractor Ex can recover a valid witness *w* from it:

(3) $$\begin{array}{c} \mathrm{Pr}[(pp,x,\omega )\in RV(x,\omega ,m)\in QV(x,\sigma ,m)=0]\approx 1 \end{array}$$

*Q* stands for all the data records (x,ω,m) that the attacker has queried so far. In other words, the attacker can forge a signature only if he really “knows” *w*.

### 2.4. Hash Time-Locked Contracts (HTLCs)

A Hash Time-Locked Contract (HTLC) is a smart contract mechanism widely used in off-chain payments, cross-chain transactions, and payment channel networks (e.g., the Lightning Network). HTLCs combine hash locks and time locks to ensure that transactions are executed securely and without the need for trust. They protect transaction execution through two locking mechanisms: hash locking and time locking [44]. A hash lock requires the recipient to provide the correct preimage to unlock the sender’s pledged funds—only those knowing x (where hash function H(x) = y) can do so. A time lock automatically returns funds to the sender if the recipient fails to provide the preimage by the deadline, preventing prolonged freezing and boosting liquidity.

**Figure 2:**
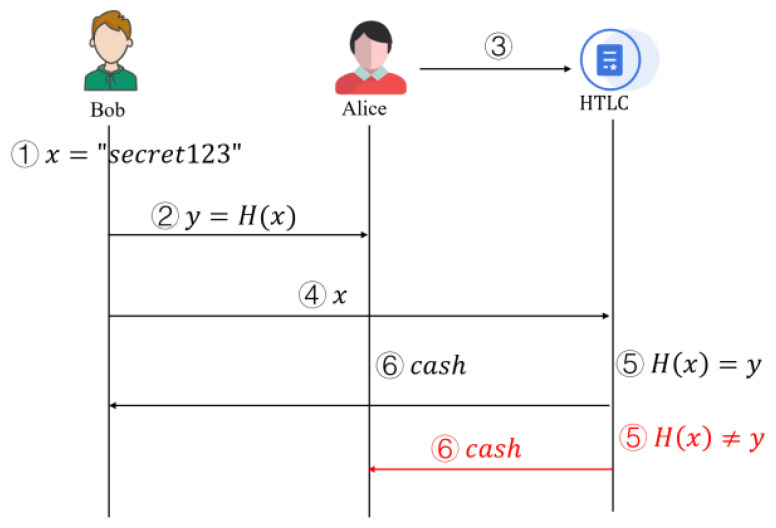
Schematic representation of HTLC.

The HTLC workflow is illustrated in detail in Figure 2: Alice wants to send cryptocurrency to Bob but wants Bob to prove that he received the payment first; HTLC allows them to make trustless payments.

**Step** **1:** Bob generates a random number (Preimage). For example, Bob chooses x= “secret123” as the secret. Bob computes its hash value H(x)=y and discloses *y* to Alice, but not *x*.
**Step** **2:** Alice creates HTLC and locks the funds. Alice deploys a smart contract, and the funds can only be unlocked in two ways: Bob provides *x* so that H(x)=y (hash lock). If Bob does not claim within *T* time, the funds are returned to Alice (time lock).
**Step** **3:** Bob collects the funds. Bob submits *x* in the contract. The smart contract verifies that H(x)=y is established, and funds are transferred to Bob.
**Step** **4:** If Bob fails to collect the funds within time *T*, Alice will get the funds back. If Bob does not submit *x* within time *T*, Alice can call the contract to retrieve her funds.

### 2.5. XMSS

XMSS (Extended Merkle Signature Scheme) is a secure post-quantum signature scheme based on hash functions. XMSS is mainly based on the Merkle tree and one-time signature (OTS) schemes, where Winternitz One-Time Signature Plus (WOTS+) is usually used as the underlying OTS. XMSS uses the Merkle tree to support multiple OTSs. Aggregating public keys supports multiple signatures without compromising security; the detailed specifics of the XMSS algorithm can be found in the literature [45].

XMSS consists of two main components, each of which is described below:

(1) WOTS+

The one-time signature scheme WOTS+ is based on a hash function, which can sign a message once, and the balance between signature size and computational efficiency is controlled by the Winternitz parameter w. The WOTS+ algorithm consists of three parts: a key generation algorithm, signature generation algorithm, and signature verification algorithm. The detailed explanation of the WOTS+ algorithm can be studied in the literature [46].

(2) Merkle tree structure

**Figure 3:**
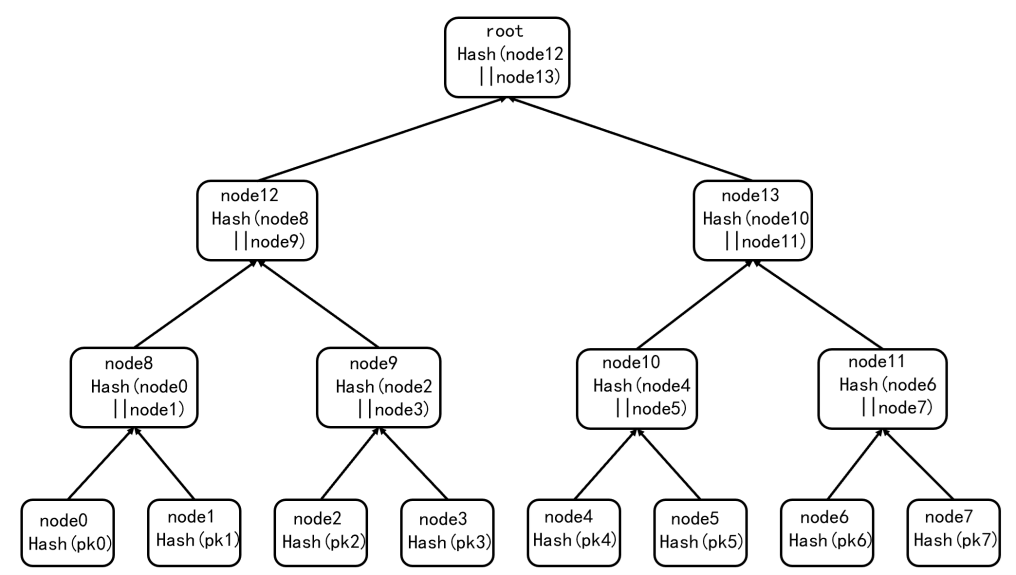
Merkle tree structure with eight leaf nodes.

Merkle leaf nodes store a hash of the WOTS+ public key. With the authentication path in the Merkle tree, a verifier can validate a signature using only the root public key instead of all individual keys. The structure of the Merkle tree is shown in Figure 3.

In the diagram above, the height of the Merkle tree is h=3, the number of leaf nodes is $2^{3}=8$, each leaf node corresponds to the placement of a WOTS+ instance, the nodes node0 and node1 are connected by a hash function to the corresponding parent node node8, the nodes node2 and node3 are connected by a hash function to the corresponding parent nodes node9, and so on until the root node is created. The root node is created.

## 3. Post-Quantum Linkable Ring Signature Scheme

Post-quantum linkable ring signature schemes based on hash functions offer notable benefits in performance, storage, and bandwidth efficiency for privacy-sensitive blockchain applications. These advantages make them especially suitable for scenarios involving high-frequency transactions. The post-quantum pluggable ring signature scheme based on hash functions is structured as follows:

### 3.1. Initialization Phase

**Figure 4:**
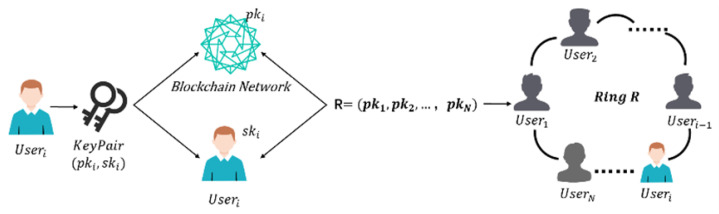
Flowchart of the initialization phase of the scheme.

The initialization phase of the process consists of two main steps, key generation and ring generation, as shown in Figure 4.

First, the security parameter λ and two hash functions are defined:

$$\begin{array}{cc} H_{k}:x_{k} & \to y_{k},H_{k}\left(1^{\lambda }\right)\to pp_{H_{k}}\\ H_{t}:x_{t} & \to y_{t},H_{t}\left(1^{\lambda }\right)\to pp_{H_{t}} \end{array}$$

Definition of knowledge signatures: $SOK_{m}\left(1^{\lambda }\right)\to pp_{s}$

(1) Key generation

(a) User-generated key pairs The user generates a key pair $\left(pk_{i},sk_{i}\right)$ using the XMSS post-quantum security scheme.

(b) Blockchain storage of public keys Users transmit their public keys $\left(pk_{i}\right)$ to the blockchain for storage.

(c) Local storage of private keys The private key $\left(sk_{i}\right)$ of the user is stored on the local device of the user. The process of key generation is shown in Algorithm 4.

(2) Cyclic manifestation

If the number of ring members is *N*, when a user needs to initiate a transaction, he chooses a ring signature group $S=\{pk_{1},pk_{2},\ldots ,pk_{N}\}$ containing himself and N−1 other users.

(a) Selection of ring signature set: The user selects a public key set $S=\{pk_{1},pk_{2},\ldots ,pk_{N}\}$ that contains himself as well as other N−1 users, which includes the user’s own public key pk and the public keys of other N−1 users.

(b) Ring signature generation: The user generates a ring signature that contains the public keys of all selected users to ensure that signatures cannot be forged.

(c) Ring signature verification: After a signature verification transaction has been initiated, other nodes verify the validity of the transaction based on the public key of the ring signature.
**Algorithm 4** Key generation.**Input:** pp**Output:** $\left(pk_{i},sk_{i}\right)$1:$sk_{i}\leftarrow \chi_{k_{i}}$2:$pk_{i}=H_{k}\left(sk_{i}\right),pk\in Y_{k_{i}}$3:**return** $\left(pk_{i},sk_{i}\right)$

### 3.2. Signature Creation Phase

(1) Selection of Signature Members: The user selects himself as a signer of the signature ring and forms an *R* ring together with other users.

(2) Calculation of Transaction Hash: Calculate the hash of the transaction content Htx=Hash(Transaction) as the basis of the signature; the ID of the transaction is *e*, the index of the signer is *j*, the public key *R* in the ring constructs a Merkle tree, rt is the root hash, the authentication path of the public key $pk_{i}$ in the Merkle tree is the path *P*, and *T* is the label, which is generated from the private key of the signer.

(3) Generation of Ring Signatures: Each signer *i* must sign the hash of the transaction with their own private key $sk_{i}$.

Using a hash function as a basis for signing allows the signer *j* to compute a ring signature based on all public keys in the ring and to determine a unique initiator from the signature values in the ring.

**Figure 5:**
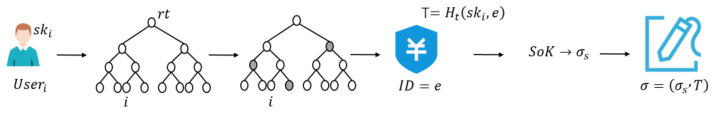
Schematic representation of the program signing phase.

The process of this stage is shown in Figure 5: the signature first constructs the Merkle tree, generates the root rt of the Merkle tree, then computes the Merkle authentication path based on the signer’s index *j*, then computes the signature label *T* based on the signer’s index and transaction ID, then generates the zero-knowledge signature $\mathrm{Sok}\sigma_{s}$, and the final output signature σ consists of the zero-knowledge signature $\sigma_{s}$ and the signature label *T*. The specific process of the algorithm can be seen in Algorithm 5.
**Algorithm 5** Signing: $\sigma \leftarrow \mathrm{Sign}(e,sk_{i},m,R)$**Input:** $e,sk_{i},m,R$**Output:** σ1:rt←Merkle(R)2:P=Path(i,merkle)3:$T=H_{t}\left(sk_{i},e\right)$4:$\sigma_{s}={\mathrm{Sok}}_{m}\cdot \mathrm{Sign}\left(pp,\left(e,rt,T\right),\left(P,i,sk_{i}\right),m\right)$5:**return** $\sigma =(\sigma_{s},T)$

### 3.3. Signature Verification Phase

#### 3.3.1. Signature Verification

Each node in the blockchain can verify the validity of a signature using the transaction details and the public key set of the signature ring. Verification using a hash function ensures that the signature is legitimate and that the transaction has not been tampered with. In the first step, the Merkle tree is computed, and in the second step, the signature is analyzed to verify zero-knowledge signatures. *T* is computed from a valid secret key $sk_{j}$ and ensures that the public key corresponding to this secret key belongs to the *R* ring.

As shown in Algorithm 6, the inputs for the signature verification phase are the event ID *e*, the signature σ, the message to be verified *m*, and the ring public key *R*. If the verification is successful, 1 is returned, indicating that the signature is valid; otherwise, 0 is returned, indicating that the signature is invalid.
**Algorithm 6** Verification: 0/1←Verify(e,σ,m,R)**Input:** e,σ,m,R**Output:** 0/11:rt←Merkle(R)2:$\mathrm{parse}\;\sigma \to \sigma_{s},T$3:**return** $0/1\leftarrow {\mathrm{SoK}}_{m}\cdot \mathrm{Verify}\left(pp,\left(e,rt,T\right),\sigma_{s},m\right)$

#### 3.3.2. Avoiding Double Spending

**Figure 6:**
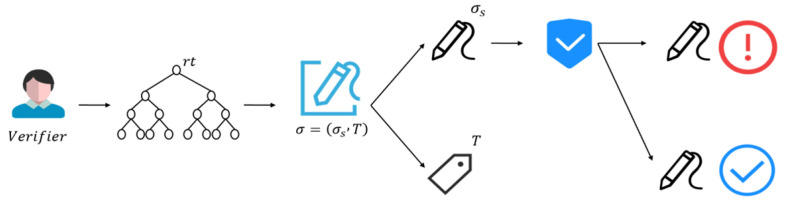
Schematic representation of the signature verification phase.

A unique ring signature is created for each transaction, which is stored along with the transaction data in the blockchain. The entire verification process is shown in Figure 6.

### 3.4. Linkability Test

Algorithm 7 ensures that transaction signatures are data-protected and that chain verifiers can recognize whether the signatures were issued by the same body.
**Algorithm 7** Linkable: $0/1\leftarrow \mathrm{link}(e,\sigma ,\sigma^{*},m,m^{*},R,R^{*})$**Input:** $e,\sigma ,\sigma^{*},m,m^{*},R,R^{*}$**Output:** 0/11:$\mathrm{parse}\;\sigma \to \sigma_{s},T$2:$\mathrm{parse}\;\sigma^{*}\to \sigma_{s}^{*},T^{*}$3:**if** $T^{*}=T$ **then**4:     **return** 15:**else**6:     **return** 0

**Figure 7:**
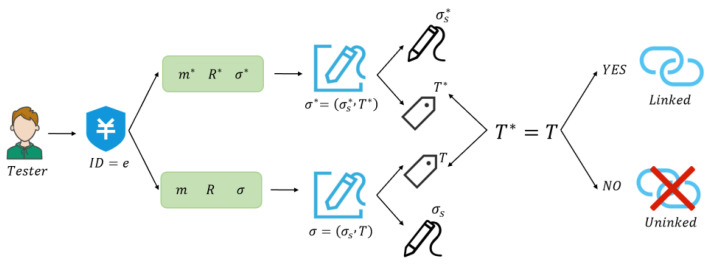
Connectivity test flowchart.

First, the transaction ID and the two signatures $\sigma ,\sigma^{*}$, the messages corresponding to the signatures $m,m^{*}$, and the ring $R,R^{*}$ are analyzed to determine whether the two signatures were generated by the same signer, and if $T=T^{*}$, the two signatures were generated with the same private key, 1, which means they can be linked (return 1, which means the two signatures were generated by the same signer); otherwise, return 0, which means they cannot be linked. This process is shown in Figure 7.

### 3.5. Overview of the Signature Scheme

Our scheme consists of four stages: initialization, signature generation, signature verification, and linkability check. In the initialization stage, the user first generates an XMSS key pair and stores the public key on the blockchain, and then completes the ring generation by selecting a set of ring signatures containing N members. In the signature generation stage, the signer constructs a Merkle tree based on the transaction hash and generates a ring signature containing a zero-knowledge proof. In the verification stage, the legitimacy of the signature is ensured by verifying the Merkle tree and the zero-knowledge signature, and a linkability check mechanism is designed to prevent double spending. In the linkability check stage, it can be verified whether the linked signatures are issued by the same entity. This scheme ensures transaction privacy while achieving signature verifiability and security against quantum attacks.

### 3.6. Security Model

The system security model is defined below in this document:

**Definition** **7** (Linkable). *A ring signature scheme is said to be linkable if a polynomial time (PPT) attacker A has a negligible probability of winning in Gamelink. Formally, the advantage (probability of success) of attacker A in Gamelink is defined as*

(4) $$adv_{A}^{link}=\mathrm{Pr}\left[A\;wins\;Gamelink\right]$$

A ring signature scheme is chainable if, for any polynomial-time attacker *A*, its advantage $adv_{A}^{link}$ is negligible (i.e., tends to zero). The chainability game ($Game_{link}$) is a game process involving a challenger *C* (Challenger) and an attacker *A* (Adversary) as follows:

**Step** **1:** Challenger generation system parameters.

Challenger *C* runs the key generation algorithm $\mathrm{pp}\leftarrow \mathrm{LRS}.\mathrm{Gen}(1^{\lambda })$, which generates the public parameter pp and sends it to attacker *A*.

**Step** **2:** Attackers attempt to forge signatures.

Predictors (oracles) that attackers can interact with include signature predictors (SO) to obtain legitimate signatures and public key registration (JO) predictors to generate and store public keys used for specific queries to limit the number of attempts by the attacker. There are three types of challenge predictors (CO).

Finally, the attacker generates *n* sets of data. $\left(e,\sigma_{i},m_{i},R_{i}\right),i\in \left[n\right]$, where *e* is public information (possibly an additional ring signature parameter), $\sigma_{i}$ is the signature, $m_{i}$ is the message, and $R_{i}$ is the set of public ring keys.

**Step** **3:** Conditions for the attacker to win the game.

Attacker *A* wins the game Gamelink under the following conditions:

(i) **Unlinkability of forged signatures:**
  Two forged signatures cannot be linked, i.e., $\mathrm{LSR}.\mathrm{link}(e,\sigma_{i},m_{i},\sigma_{j},m_{j},R_{i},R_{j})=0$. This means that the signatures $\sigma_{i}$ and $\sigma_{j}$ were generated using the same secret key, but the system is unable to link them, ensuring unlinkability.
(ii) **Validity of the forged signature:**
  The signature must be valid, i.e., $\mathrm{LSR}.\mathrm{Verify}(e,\sigma_{i},m_{i},R_{i})=1$, meaning the forged signature $\sigma_{i}$ must pass the verification algorithm.
(iii) **Signature is not obtained from the signing oracle (SO):**
  The attacker must generate the forged signature independently, rather than simply copying one returned by the signing oracle SO.
(iv) **Restricted public key usage:**
  All public keys must be registered through the public key registration predicate JO, which ensures that the attacker can only use public keys that have been properly registered and cannot introduce external keys.
(v) **Limited access to the challenge predictor (CO):**
  The attacker can only query the challenge predictor CO a limited number of times, preventing brute-force attempts or exhaustive exploration of the input space.

Chainability requires that an attacker cannot use the same private key to generate two signatures that cannot be correlated, i.e., if a user uses the same private key multiple times for signatures, these signatures should be able to be linked to each other; otherwise, the attacker can evade the correlation and achieve malicious behaviors such as double payments.

**Definition** **8** (Anonymity). *The advantage (probability of success) of attacker A in $game_{anon}$ is defined as follows:*

(5) $$adv_{A}^{anon}=\mathrm{Pr}\left[A\;wins\;Gameanon\right]$$

*A ring signature method is considered anonymous for any attacker A in polynomial time (PPT) if its advantages are satisfied:*

(6) $$adv_{A}^{anon}\leq \frac{1}{n}+\mathrm{negligible}$$

*In other words, an attacker cannot identify the signer more efficiently than with a random estimate. In this case, 1/n indicates the success rate of a purely random estimate, and “negligible” means that the additional probability of success is negligible even if the attacker has some computing power.*

Anonymous $game_{anon}$ is a game process between challenger C (Challenger) and opponent A (Adversary). The flow of the game is as follows:

**Step** **1:** Challenger generation system parameters.

Challenger *C* first runs the $\mathrm{pp}\leftarrow \mathrm{LRS}.\mathrm{Gen}(1^{\lambda })$ key generation algorithm to generate the public parameter pp and sends it to attacker *A*.

**Step** **2:** The attacker chooses the signature scenario

*A* chooses an event identifier *e* (which can be used to mark a transaction or an identity), a message *m* (which must be signed), and a set of ring public keys $R={\left\{{\mathrm{pk}}_{i}\right\}}_{i\in [n]}$, where *R* is generated by the attacker via a public key predicate (JO) registration engine by generating r←AOJ(pp), and *A* sends (e,m,R) to the challenger *C*.

**Step** **3:** The challenger randomly selects a signer and creates a signature.

The challenger *C* first chooses an index *b* at random: b←$[n], where *b* denotes the position of the selected signer in the ring. *C* then generates a ring signature with the secret key ${\mathrm{sk}}_{b}$ of this signer, $\sigma \leftarrow \mathrm{LRS}.\mathrm{Sign}(e,{\mathrm{sk}}_{b},m,R)$, and *C* sends the signature σ to the attacker *A*.

**Step** **4:** The attacker attempts to forge the signature associated with the signer.

The attacker *A* uses the resources of Signature Overtaking Machine (SO), Public Key Registration Overtaking Machine (JO), and Challenge Overtaking Machine (CO) and generates a new signature: $\left(\sigma^{\prime },m^{\prime },R^{\prime }\right)\leftarrow \mathrm{ASO},\mathrm{CO},\mathrm{JO}\left(\mathrm{pp},\sigma \right)$, where $\sigma^{\prime }$ is the new signature forged by the attacker, $m^{\prime }$ is the message corresponding to the forged signature, and $R^{\prime }$ is the set of public keys for the forged signature.

**Step** **5:** Conditions for the attacker to win the game.

Attacker A wins the game $game_{slan}$ under the following conditions:

(1) The target public key pk cannot have been queried by the CO or the SO: this ensures that an attacker cannot obtain a legitimate signature by direct query.

(2) The forged signature $\sigma^{\prime }$ must not come from the SO predicate: this prevents the attacker from simply repeating a valid signature obtained from SO, but actually forging a new signature.

(3) The forged signature must be verified: $\mathrm{LRS}.\mathrm{Verify}(e,\sigma^{\prime },m^{\prime },R^{\prime })=1$ means that the signature generated by the attacker looks like it was generated by the private key of one of the ring members.

(4) The forged signature must be linked to the signature generated by the challenger:

$\mathrm{LRS}.\mathrm{Link}(e,\sigma ,\sigma^{\prime },m,m^{\prime },R,R^{\prime })=1$

Non-defamation requires that an attacker cannot generate a signature so that it appears to be linked to an honest user’s signature (i.e., an attacker cannot forge a signature to plant an honest user). In other words, an attacker cannot create a signature that is considered to be generated by the same private key as a legitimate signature in a linkability check.

In this paper, the scheme combines Merkle tree proof of public key membership to avoid traversing the public key in the ring and improve efficiency, and uses zero-knowledge proof (ZKP) to protect the identity of the signer and ensure anonymity. Meanwhile, linkability detection is performed by signature tag T to prevent double-signature attacks. The scheme uses hash computation instead of traditional large-number operations and has the characteristics of high efficiency, security (anti-quantum attack, based on the hash function), anonymity (ring signature guarantees the anonymity of the signer), and linkability (can detect duplicate signatures of the same private key), making it suitable for scenarios of anonymous transactions in blockchain, privacy-protecting authentication, and post-quantum secure signatures.

## 4. Application

This scheme is designed for a privacy-preserving off-chain payment network, which ensures quantum resistance, anonymity, prevention of double payments, and efficient payment settlement of the scheme. The specific details are as follows: An XMSS ring signature is used as the payment authentication method to resist quantum computing attacks, thus realizing post-quantum security. The ring signature and Onion Routing are used to hide the payment path and prevent transaction correlation analysis (enhance anonymity). A linkable ring signature (LRS) is constructed to ensure payment uniqueness (prevent double payments). Hash Time Lock Contract (HTLC) and Zero Knowledge Proof (ZKP) are combined to optimize off-chain payment rates (efficient payment settlement).

### 4.1. Participants

The participants in the scheme model of blockchain off-chain payments include payers, receivers, relay nodes, and smart contracts, and the following is a description of each participant’s responsibility function:

(1) Payer (*S*): Responsible for payments outside the chain and wishes to conceal his or her identity.

(2) Recipient (*R*): After receiving payment, the recipient chains and processes the transaction.

(3) Relay node (A,B,C,…): It is responsible for forwarding payment routes and cannot identify the payer and recipient.

(4) Smart Contracts (SC): Responsible for dispute resolution and final settlement of funds on the main chain.

### 4.2. Key Technologies

(1) XMSS linkable ring signature

XMSS is a post-quantum stateless signature scheme based on hash functions that can be used to create ring signatures and ensure anonymity and anti-quantum security. During the payment process, the payer *S* chooses a ring *R* (which contains itself and the n−1 random public key of a user) to generate a ring signature that ensures anonymity and linkability:

Anonymity: The system ensures that adversaries cannot identify the actual payer in a transaction.

Linkability: The protocol prevents double-spending attempts by the same user employing identical cryptographic keys.

The implementation of a ring signature linkable to XMSS is described below, and the parameters of the XMSS linkable ring signature scheme are listed in Table 1.

The algorithm of the scheme mainly includes key pair generation (Algorithm 8), signature generation (Algorithm 9), signature verification (Algorithm 10) and linkability testing (Algorithm 11), which are described below.

(a) Key Generation

The algorithmic inputs to the XMSS linkable ring signature key pair generation algorithm are the height *h* of the Merkle tree as well as the private key sk. A tree height of *h* means that the tree consists of a total of $2^{h}$ leaf nodes, i.e., the ring is made up of $2^{h}$ members, and the private key sk is made up of $2^{h}$ one-time signature algorithms of the private key $x_{i}$, where $i\in [0,2^{h}-1]$.
**Algorithm 8** Public key generation.**Inputs:** *h*, SK**Output:** PK1:$node_{i}=Hash(node_{2i+1}||node_{2i})$, $i\in [0,2^{h}-1]$2:Root=Hash(node1||node2)3:PK=Root

(b) Signature

The inputs to the algorithm are the message digest *M*, the hash function *H* and the one-time signed key pairs $\left(x_{i},y_{i}\right)$, $i\in [0,2^{h}-1]$. During the signing process, the node with index *i* and the key pair $\left(x_{i},y_{i}\right)$ that corresponds to the unique signature of node *i* are selected first. The message is then signed once to generate the unique signature $\sigma_{\mathrm{OTS}}$ and the authentication path $auth_{i}$ is computed, which corresponds to the public key $y_{i}$ of the unique signature. The final signature is $\sigma =(i,\sigma_{\mathrm{OTS}},Y_{i},auth_{i})$.
**Algorithm 9** Creating the signature.**Inputs:** *M*, *H*, one-time signature key pair $\left(x_{i},y_{i}\right)$**Output:** σ1:$\left(x_{i},y_{i}\right)$, $i\in (0,2^{h}-1)$2:**for** $x_{i}$ **do**3:  Select node $x_{i}$, digitally sign the message *M* once and generate the corresponding signature $\sigma_{\mathrm{OTS}}$4:     Calculate the authentication path for $y_{i}$5:$\sigma =(i,\sigma_{\mathrm{OTS}},Y_{i},auth_{i})$

(c) Signature verification

The verification part is divided into two parts; both parts are to verify the correctness of the one-time signature, and the second part is to reconstruct the root node of the Merkle tree according to the node i selected by the signature, and then compare the root with the value PK of the public key of PK XMSS. If they are the same, the verification is passed. Both parts of the validation must be correct.
**Algorithm 10** Signature verification.**Inputs:** σ**Output:** true or false
1:**if then**2:    $\mathrm{VER}(M,\mathrm{sig}\left(\mathrm{OTS}\right),Y_{i})=\mathrm{true}$3:    Reconstruct the root node root’ of the Merkle tree using *i* and $Y_{i}$4:    **if** ${\mathrm{Root}}^{\prime }=\mathrm{PK}$ **then**5:         **return** true6:    **else**7:         **return** false                   ▹ Signature is invalid8:**else**9:    **return** false

(d) Verification of linkability

This step first needs to notate the *i*, $Y_{i}$, $auth_{i}$ in $\sigma =(i,\sigma_{\mathrm{OTS}},Y_{i},auth_{i})$ as label T. Another set of information about the message, signature, and ring is also needed for linkability checking, i.e., $m^{*}$, $\sigma^{*}$ and $R^{*}$, $\sigma_{\mathrm{OTS}}$ will be written as $\sigma_{s}$, then $\sigma_{s}^{*}$ denote $\sigma_{\mathrm{OTS}}^{*}$, the primary process of verification is to compare the values of $T^{*}=T$ are the same or not, respectively, and if the labeling information is the same, then it is proved that the signatures were indeed made by the same signer.
**Algorithm 11** Linkable $0/1\leftarrow link(e,\sigma ,\sigma^{*},m,m^{*},R,R^{*})$**Inputs:** $e,\sigma ,\sigma^{*},m,m^{*},R,R^{*}$**Output:** 0/11:$parse\;\sigma \to \sigma_{s},T$2:$parse\;\sigma^{*}\to \sigma_{s}^{*},T^{*}$3:**if** $T^{*}=T$ **then**4:     **return** 15:**else**6:     **return** 0

(2) Onion Routing

Each relay node can decrypt only the transaction information of its immediate neighbors and does not have knowledge of the entire payment path, thereby ensuring path privacy.

With hop-by-hop encryption, the relay node can only see that the sender is the previous node. The receiver is the next node. Encrypted information includes the transaction amount and expiration date.

(3) Hash Time-Locked Contracts (HTLCs)

HTLCs are mainly based on two locking mechanisms: hash lock and time lock. HTLCs allow payers to make conditional payments in an off-chain network using hash commitments. The payer first generates *S*, a secret value *r* and then computes a hash value H(r) of the secret value. The means of payment are locked in the smart contract and the recipient *R* can only unlock the payment by providing *r* within a certain period of time. If the lock is not lifted at the end of the time period, the funds are returned to the payer.

(4) Zero-Knowledge Proof (ZKP)

Using ZKP to validate payment channels prevents payment details from being made public:

Ring signature verification: The recipient (*R*) can use ZKP to verify whether the originator is a member of the ring without revealing his or her identity.

Payment channel confidentiality: The use of zk-SNARKs to ensure that relay nodes cannot correlate transaction sources.

### 4.3. Transaction Process

**Figure 8:**
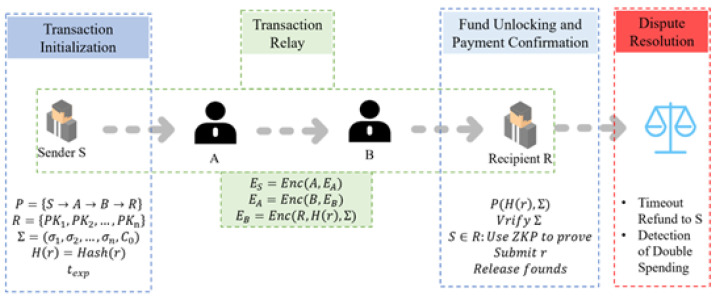
Flowchart of an exemplary application of the program.

The whole transaction process is divided into four stages. The first stage is the transaction initialization; this stage mainly has the payer *S* choose the payment route, select the ring members, generate the corresponding ring signature, and set the timeout time. The second stage is the transaction relay, which functions in accordance with the transaction path to complete the transmission of encrypted information. The payer, the receiver, and the relay nodes *A* and *B* will be involved in the third stage, which is the fund unlocking and payment confirmation phase. This phase is mainly completed by the receiver; the receiver needs to verify the correctness of the ring signature, verify that the sender *S* really belongs to the ring *R*, and release the funds. The fourth phase is the evaluation of the payment timeout and the double payment phenomenon, and the flow of the whole phase can be seen in the following Figure 8:

(1) Transaction initialization

The payer *S* chooses the payment path P={S→A→B→R}, i.e., *S* is passed to *A*, which passes to *B*, which is given to the final recipient *R*. The payer *S* chooses the ring-signing public key. The set $R=\{PK_{1},PK_{2},\ldots ,PK_{n}\}$ contains itself and other n−1 random users. The XMSS ring signature is generated as follows: $\Sigma =(\sigma_{1},\sigma_{2},\ldots ,\sigma_{n},C_{0})$. Next, the hash commitment value H(r)=Hash(r) is computed, the HTLC transaction is constructed, and the timeout time $t_{\exp}$ is set.

(2) Transaction relay

**Figure 9:**
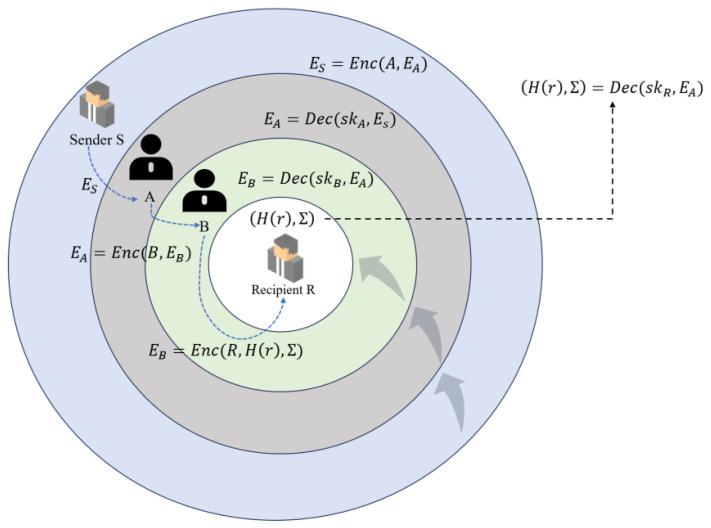
Transaction diagram of nodes in the Onion Routing structure.

Before the transaction sending starts, sender *S* encrypts the payment message using Onion Routing: ES=Enc(A,EA), EA=Enc(B,EB), EB=Enc(R,H(r),Σ). The sender *S* sends the encrypted message ES to the relay node *A*. The relay node receives ES and then decrypts it using its own private key $sk_{A}$ to get the EA, but *A* has no way of knowing the encrypted message of the EA. *A* only knows that it received the payment from *S* and it needs to be forwarded to *B* so that the relay node *A* sends EA=Enc(B,EB) to relay node *B*; similarly, relay node *B* decrypts it using its own private key $sk_{B}$ to get EB, but *B* cannot learn the encrypted information of EB, and *B* only knows that it received the payment from *A* and it needs to be forwarded to the receiver *R*. So the relay node *B* will send $E_{B}=\mathrm{Enc}\left(R,H\left(r\right),\Sigma \right)$ to the receiver *R*. The specific process can be seen in Figure 9 below.

The relay nodes in this process perform hop-by-hop decryption, but have no way of knowing the source of the payment. *A* only knows that it received the payment from *S* and forwards it to *B*. *B* only knows that it received the payment from *A* and forwards it to *R*. Such a process adequately guarantees the privacy of the path.

(3) Fund unlocking and payment confirmation

In this phase, the receiver *R* decrypts EB to obtain the hash commitment value H(r) as well as the post-quantum ring signature Σ. Next, the receiver checks whether Σ is valid using the ring signature verification algorithm, XMSSVerify(m,R,Σ)=True, and then uses zero-knowledge proof (ZKP) to verify that the sender *S* is indeed a member of the ring *R*, i.e., uses ZKP to prove that the ring signature comes from one of the users in the ring without revealing the identity of the payer. The receiver *R* decrypts and generates *r* and unlocks the HTLC funds: SC→ReleasefundstoR.

(4) Dispute resolution

If a timeout occurs, that is, the $t_{\exp}$ expires, the HTLC automatically reimburses the payer *S*. Linkable ring signatures also prevent *S* from making duplicate payments with the same key, i.e., recognizing duplicate payments.

**Figure 10:**
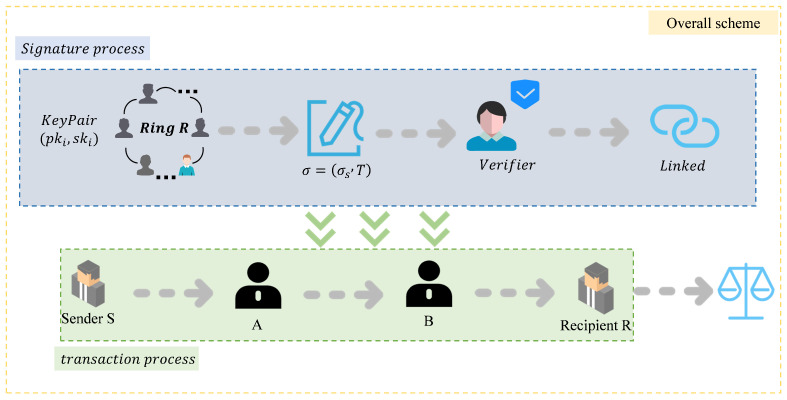
Overall scheme.

Our overall solution framework is shown in Figure 10.

## 5. Security Analysis

### 5.1. Security Assessment

In this system, XMSS ensures the resilience of the payment system to quantum attacks, ring signatures, and Onion Routing, and KP provides strong anonymity; HTLC and zero-knowledge proofs increase the speed of settlement and enable efficient off-chain settlement; linking the possible ring signature ensures the uniqueness of each settlement and prevents settlement duplication problems, particularly in the following aspects.

(1) Anonymity: Ring signatures conceal the payer’s identity, ensuring that even if transactions are recorded on-chain, the real user remains untraceable. Onion Routing further guarantees that relay nodes cannot observe the complete payment path.

(2) Quantum Resistance: XMSS, being hash-based, resists quantum computing attacks. The HTLC employs post-quantum hash functions (e.g., SHAKE256) to maintain security.

(3) Resistance to On-Chain Analysis: Even when transactions are on-chain, the combination of ring signatures and zero-knowledge proofs (ZKPs) prevents adversaries from linking payers and recipients.

(4) Computational and Storage Optimization: While XMSS signatures are relatively large, their parameters can be optimized (e.g., via compact modes). Payment channels cache Merkle authentication paths to reduce computational overhead.

### 5.2. Threat Model

Before formally proving the security of the scheme of this paper, it is necessary to identify the types of attacks that may be received, i.e., considering the capabilities of the hypothetical attacker and the possible ways of attacking, there are the following possibilities:

(1) Passive Adversaries: These attackers observe on-chain data to analyze transaction patterns and trace payment paths.

(2) Active Adversaries: They may control a subset of relay nodes and attempt to compromise anonymity or forge signatures.

(3) Quantum Adversaries: Possessing quantum computing capabilities, such attackers can execute Shor’s algorithm to break public key encryption schemes.

(4) Double-Spending Adversaries: These attackers attempt to bypass the HTLC mechanism by exploiting multiple signatures to achieve double payments.

### 5.3. Security Proofs

**Theorem** **1.** *The linkable ring signature (LRS) designed in this paper is linkable under the Random Oracle Model (ROM) provided that the zero-knowledge proof (SoK, Statement of Knowledge) scheme relied upon satisfies correctness, simulatability, and simulation extractability, while the underlying hash function Hk has one-wayness and collision resistance (collision resistance).*

**Proof.**  Assuming there exists an adversary *A* who can win the linkability game (see Definition 7) with non-negligible probability, we can construct an emulator *S* that breaks either the one-wayness (preimage resistance) or the collision resistance of the underlying hash function $H_{k}$. Specifically, one-wayness is broken by requiring that, given a hash output $h_{o}$, an input *x* is found such that $h_{o}=H_{k}\left(x\right)$; collision resistance is broken by requiring that, given an input *x*, a distinct input $x^{\prime }$ is found such that $H_{k}\left(x\right)=H_{k}\left(x^{\prime }\right)$. If *A* is able to successfully forge a set of ring signatures that are not linkability-independent, then *S* can leverage this ability to break the hash function. □

Detailed proof follows in three main stages:

(1) Initialization of the *S* simulator

The simulator *S* needs to break the one-way or collision-resistant nature of the hash function. So at the start of the game, it is given a one-way challenge instance ho (which needs to find the preimage *x*), and a collision-resistant challenge instance xc (which needs to find the collision input $x^{\prime }$). *S* then generates the public parameter pp and sets the hash ${\hat{H}}_{t}$ to a randomized preimage machine.

(2) Processing queries

Attacker *A* can interact with the system to make the following queries:

(A) Prophecy Machine 1: Join Oracle (JO(⊥)) *A* can request to join the ring-signing system to obtain a public–private key pair (pk,sk). *S* randomly selects two indexes when processing the $q_{j}$ join queries proposed by *A*. *A* can request to join the ring-signing system to obtain a public–private key pair (pk,sk).
  (a) One index is used to challenge one-wayness ($q_{j}^{o}$): this returns $pk_{o}=ho$.
  (b) One index is used to challenge collision resistance $q_{j}^{c}$: at this point, it returns $pk_{c}=H_{k}\left(x_{c}\right)$ and sets the private key $sk_{c}=x_{c}$.
(B) Prophecy Machine 2: Corruption Oracle ($CO(pk_{i})$) Allow *A* to query the private key corresponding to a specific public key $pk_{i}$. If $pk_{i}=pk_{o}$, *S* terminates the game directly (to avoid exposing instances of cracking one-wayness). If $pk_{i}=pk_{c}$, *S* returns $sk_{c}=x_{c}$. In other cases, *S* typically returns the private key.
(C) Prediction machine 3: Signature Oracle ($SO(R,e,pk_{i},m)$) *A* may request a signature for message *m*. *S* needs to simulate the signature generation process. If the signer’s public key $pk_{i}\neq pk_{o}$, then generate the ring signature σ in the normal way. If $pk_{i}=pk_{o}$, then *S* generates a simulatable signature using the zero-knowledge proof simulator Sim and records the hash table entries. Randomized Prophecy Machine Query ($H_{t}\left(x,e\right)$): If *A* has previously queried (x,e), return the recorded value. If $H_{k}\left(x\right)=pk_{o}$ and there is already a relevant entry in the hash table, return *x* as the answer to the one-wayness challenge. Otherwise, return a random value.

(3) Challenge phase

In the challenge phase, *A* must produce *n* unlinkable ring signatures: ${\left(e,\sigma_{i},m_{i},R_{i}\right)}_{i\in [n]}$ (each signature $\sigma_{i}=\left(\sigma_{s,i},T_{i}\right)$). Since *A* can perform CO queries for at most n−1 keys, this means the following:

(A) There is at least one $sk^{*}$ key whose public key has never been queried for CO or has a key $sk^{*}$ whose public key has indeed been queried for CO, but the returned key is different from $sk^{*}$.

(B) Denote $pr_{A}^{1}$ and $pr_{A}^{2}$, respectively, the probability that *A* wins in these two ways, satisfying $pr_{A}^{1}+pr_{A}^{2}={\mathrm{adv}}_{A}$.

(a) Case 1 (cracking the one-way street)

If $pk^{*}=pk_{o}$, then the key $sk^{*}$ needs to satisfy $H_{k}\left(sk^{*}\right)=h_{o}$, which is precisely the one-way challenge of the hash function, so *S* can use it to break one-wayness.

(b) Case 2 (unidirectional cracking)

If $pk^{*}=pk_{c}$, then the attacker finds two different private keys $sk^{*}\neq sk^{\prime }$ such that $H_{k}\left(sk^{*}\right)=H_{k}\left(sk^{\prime }\right)$. This is exactly the collision resistance challenge of the hash function, so *S* can use it to break the collision resistance. In both cases, *S* succeeds in constructing a cracking instance with success probability:

$\frac{q_{j}-q_{r}}{q_{j}}\cdot \frac{1}{q_{j}-d+1}\cdot pr_{A}^{\left(1\right)}$ (for one-wayness) and $\frac{q_{j}-q_{r}}{q_{j}}\cdot \frac{1}{q_{j}}\cdot pr_{A}^{\left(2\right)}$ (against collision resistance) are non-negligible and thus contradictory.

Since S can break either one-wayness or collision resistance by a successful attack on A, and both properties are assumed to be secure (unbreakable), A cannot successfully break the linkability of the LRS. This proves that our linkable ring signature scheme is linkable under the stochastic predicate machine model.

**Theorem** **2.** *The linkable ring signature system (LRS) proposed in this paper is anonymous according to the randomized predicate machine (ROM) model.*

**Proof.**  Anonymity requires that given a ring *R* and a signature σ, the attacker *A* cannot distinguish which public key pk∈R generated the signature. That is, the probability that an attacker wins the **Anonymity Game (Definition 8)** cannot significantly exceed the probability of a random guess 1/n (where *n* is the size of the ring). Assuming that an *A* exists that can win the anonymity game with non-negligible probability ${\left\{\mathrm{adv}\right\}}_{A}$, we construct the simulator *S* such that *A* is unable to distinguish between authentic signers. □

(1) Setting the publication parameters *S* runs the key generation algorithm $pp\leftarrow \mathrm{LRS}.\mathrm{Gen}(1^{\lambda })$ to generate the public parameters and send them to *A*. The hash functions ${\hat{H}}_{k}$ and ${\hat{H}}_{t}$ are modeled as random oracles, so that *S* must simulate these oracles at query time.

(2) Processing queries

(a) Join Oracle (JO): *A* can request to join a signature ring system to receive a public key pk; *S* directly takes a random public key pk and sends it back to *A*. The key here is that *S* does not need to generate a private key, which helps the simulation process afterwards.

(b) Random querying of the predicate (${\hat{H}}_{k}\left(x\right)$ and ${\hat{H}}_{t}\left(x\right)$): If *A* has previously queried the same input *x*, the stored value is returned. If it is a new query, $y\in Y_{k}$ is randomly selected and stored in a hash table.

(3) Challenge phase

In the challenge phase, the following occurs:

(A) *A* selects a ring $R={\left\{pk_{i}\right\}}_{i\in [n]}$ with *n* public keys, event ID *e* and message *m* and sends (R,e,m) to *S*.
(B) *S* creates a challenge signature, which consists of the following six steps:
  (a) Construct the Merkle root rt (computed from the set of public keys *R*).
  (b) Random selection of marker $T∼Y_{t}$.
  (c) Randomly select an index b∼[1,n] that specifies the position of the challenge signer, i.e., public key *b*, as the signer.
  (d) Calculate x={rt,T,e,m}.
  (e) Zero-knowledge test simulator: First, we run $\mathrm{SimG}\left(1^{\lambda }\right)\to \left(pp,\tau \right)$, then SimS(pp,τ,x)→tr (to generate the SoK proof of the simulation).
  (f) Generate a control signature: σ=(tr,T)
  (g) Send the signature σ to *A*.
(C) *A* tries to guess $b^{\prime }$: *A* must guess whether $b^{\prime }$ is equal to *b*. If *A* can determine *b* better than a random value (probability 1/n), the attack is successful.

Due to the simulatability of zero-knowledge evidence, the fact that the signature does not depend on the secret key, the randomly chosen tag *T*, and the randomly chosen index *b*, the probability that an attacker can successfully identify the signer is $pr\left[b^{\prime }=b\right]=\frac{1}{n}$. This corresponds to the probability of a random guess and indicates that an attacker cannot obtain any additional information.

Since *A* cannot win the anonymity game with a probability significantly greater than 1/n, the linkable ring signature procedure is shown to be anonymous according to the probabilistic predicate machine model.

**Theorem** **3.** *The linkable ring signature (LRS) scheme has Non-Slanderability under the randomized predicate machine (ROM) model, i.e., an attacker A cannot forge a signature such that it points to some public key pk that has not actually signed the message, i.e., A cannot maliciously construct a signature to trap an unsigned user.*

**Proof.**  An attacker in the Non-Slanderability model needs to conduct the following:

(1) Construct a legitimate signature $\sigma^{\prime }$ such that $\mathrm{LRS}.\mathrm{Verify}(e,\sigma^{\prime },m^{\prime },R^{\prime })=1$ (successful verification).

(2) However, the pk must not have been previously used for signature search (SO) or key search (CO).

(3) If *A* successfully forges the signature, *A* can be used to construct a simulator *S* that breaks the one-wayness of the hash function.

Assuming that *A* can win the defeasibility game with non-negligible probability, construct a simulator *S* that can break the one-wayness of the hash function ${\hat{H}}_{t}$. □

**Step** **1:** Setting the publication parameters.

First, the public parameters are generated by *S*: run $pp\leftarrow \mathrm{LRS}.\mathrm{Gen}(1^{\lambda })$ and send it to *A*. Then, perform simulations of the stochastic predictors ${\hat{H}}_{k}$ and ${\hat{H}}_{t}$: ${\hat{H}}_{k}$ is a stochastic predictor, which can be programmed, and ${\hat{H}}_{t}$ is also a stochastic predictor, which we would like to utilize in order to break one-wayness.

**Step** **2:** Processing queries.

To simulate the whole game environment, *S* needs to process *A*’s queries, including the following:

(1) Join query (JO): When *A* queries JO(⊥), *S* randomly selects the public key and returns it.

(2) Key query (CO): When *A* queries $CO(pk_{i})$, *S* randomly selects the private key $sk_{i}$, and *S* also programs a randomized predicate machine ${\hat{H}}_{k}$ such that $pk_{i}={\hat{H}}_{k}\left(sk_{i}\right)$; in addition to this, *S* records $sk_{i},pk_{i}$ to a hash table.

(3) Signature query (SO): *A* provides a public key pk∈R, a message *m* and an event ID *e*. If pk has been used in a CO query, *S* generates a signature using the standard LRS.Sign, and if pk has not been queried by a CO query, then *S* generates a randomized private key $\hat{sk}$, programs ${\hat{H}}_{k}$ such that $pk={\hat{H}}_{k}\left(\hat{sk}\right)$, generates a signature using LRS.Sign, and records $\hat{sk},pk$ to the hash table.

(4) Randomized Predictor Query ${\hat{H}}_{k}\left(x\right)$ and ${\hat{H}}_{t}\left(x,e\right)$: If *A* queries ${\hat{H}}_{k}\left(x\right)$, *S* checks the hash table; if *x* already exists, it returns the corresponding value; if *x* does not exist, it randomly samples *y* and stores it in the hash table; if *A* queries ${\hat{H}}_{t}\left(x,e\right)$, *S* checks if the record (·,pk,(e,T)) exists such that ${\hat{H}}_{t}\left(x,e\right)=T$. Then *S* directly returns (x,e) as an answer to the challenge of cracking the one-wayness; otherwise, it randomly samples *y* and stores it in the hash table.

**Step** **3:** Challenge phase.

The challenge stage is divided into three parts:

(1) *A* selects the challenging parameters:

A public key set *R*, some public key pk, a provision message *m* and an event ID *e*, where pk cannot appear in a CO or SO query.

(2) *S* generates the challenge signature:

Firstly, *S* sets the hash challenge target T=ho, then computes x={rt,T,e,m}, where rt is the *R*-generated Merkle root, and then generates tr using the SoK simulator: $\mathrm{SimG}\left(1^{\lambda }\right)\to \left(pp,\tau \right)$, SimS(pp,τ,x)→tr. After that, it generates the signature σ=(T,tr), and finally records pk,(e,T) to the hash table.

(3) *A* generates forged signatures:

*A* generates a set of public keys $R^{\prime }$, messages $m^{\prime }$ and signatures $\sigma^{\prime }=\left(\sigma_{s}^{\prime },T^{\prime }\right)$, which need to satisfy $\mathrm{LRS}.\mathrm{Verify}(e,\sigma^{\prime },m^{\prime },R^{\prime })=1$ and pk has not been queried by CO or SO.

**Step** **4:** Critical analysis.

(1) Simulation extractability:

According to the simulation extractability property of SoK, the private key ${\hat{sk}}^{\prime }$ can be extracted from $\sigma^{\prime }$ such that $T^{\prime }={\hat{H}}_{t}\left({\hat{sk}}^{\prime },e\right)$. Since $\mathrm{LRS}.\mathrm{Link}(e,\sigma ,\sigma^{\prime },m,m^{\prime },R,R^{\prime })=1$, it follows that $T^{\prime }=T=ho$.

(2) Bypassing the one-way hash function:

Given that *A* provides a value ${\hat{sk}}^{\prime }$ such that ${\hat{H}}_{t}\left({\hat{sk}}^{\prime },e\right)=ho$, the simulator *S* can return $\left({\hat{sk}}^{\prime },e\right)$ as a solution to the one-wayness challenge of the hash function.

Thus, a simulator *S* that breaks the one-wayness of the hash function is successfully constructed, which contradicts the assumption that the hash function ${\hat{H}}_{t}$ is one-way. Therefore, under the assumption that ${\hat{H}}_{t}$ is one-way, the attacker *A* cannot win the unforgeability game with non-negligible probability. Consequently, the proposed LRS scheme is non-slanderable.

**Theorem** **4** (Unforgeability). *An LRS scheme is unforgeable if it satisfies the following properties:*

*(1) Linkability: An attacker cannot forge a signature to bypass link detection.*

*(2) Non-Slanderability: An attacker cannot maliciously construct a signature to trap an innocent user.*

**Proof.**  Since Theorem 3 proves that the scheme is not falsifiable, it follows that the LRS scheme is unforgeable when combined with linkability. □

## 6. Performance Analysis

In this section, the performance of the method presented here is analyzed and compared with that of methods in the literature. The primary operations of this signature scheme encompass the generation of public and private keys, the measurement of time required for message signing and verification, and instantiation with SHA-256 as the underlying hash function. This scheme utilizes XMSS-SHA2-10-256 as the digital signature algorithm, which is based on the SHA-256 hash function and features a Merkle tree height of 10, thereby supporting up to $2^{10}=1024$ ring signatures. First, the computational efficiency in terms of key generation, signature, and verification time is analyzed. Then, the functional and security aspects are compared to highlight the advantages of this method in an off-chain blockchain payment scenario.

### 6.1. Signature Efficiency Analysis

(1) Key generation time

Since much of the literature does not provide information on the key generation time, we mainly focus on comparable methods. According to the information provided in Table 2, the key generation time of the method presented here is 2.06 s, which is quantitatively not directly comparable with the literature based on the same hash function [18]. However, compared with the literature based on STARK [25] and the literature based on the lattice [26], the latter has a higher signature and verification overhead, which suggests that the key generation step may be more complex.

(2) Signature time

The signature time of the method presented here is 1.97 s, which means a higher signature overhead than the STARK-based literature [25] (160 ms) and the hash function-based literature [18] (0.06 s). This is mainly due to the fact that the method in this work uses hash function-based ring signatures in combination with zk-SNARKs to improve privacy, which increases computational complexity.

(3) Verify time

The verification time of the method in this work is $9.47\times 10^{-4}$ s, which is much shorter than [25] (112 ms), [18] (0.06 s) and [26] (3584 ms). This result shows that the method in this work has a very low computational cost in the signature verification phase and is suitable for blockchain application scenarios that require fast verification.

**Figure 11:**
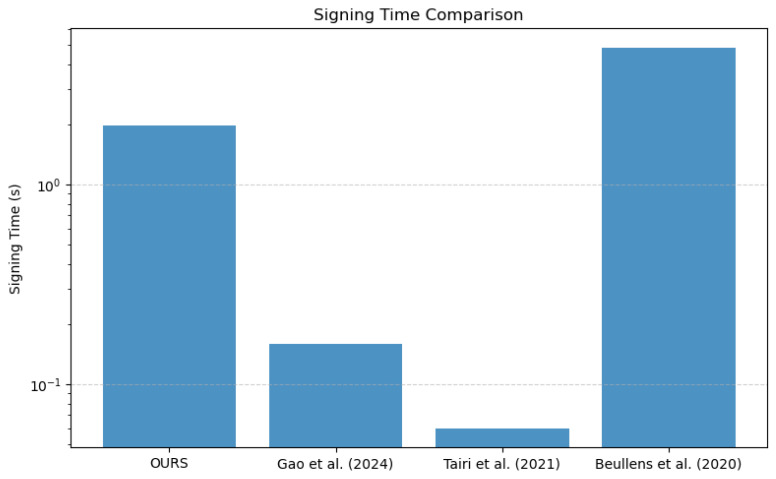
Signature time comparison diagram, [18,24,26].

**Figure 12:**
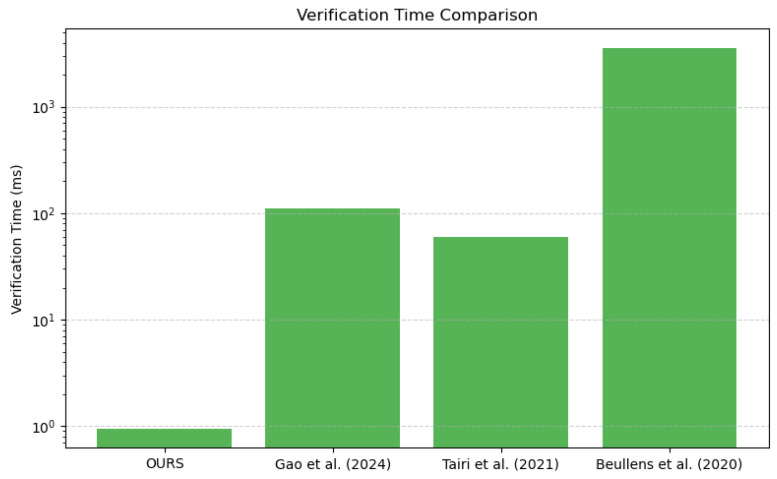
Signature verification comparison diagram, [18,24,26].

Figure 11 and Figure 12 more intuitively illustrate the performance of our scheme in terms of signature time and verification time. Overall, while the proposed scheme is slightly less efficient than some existing schemes in terms of signature time, it demonstrates a substantial advantage in verification efficiency, rendering it especially suitable for rapid transaction confirmation scenarios in blockchain applications.

The proposed scheme achieves a significant improvement in verification efficiency and enhanced privacy at the cost of a slightly higher signing time, making it particularly suitable for distributed systems that prioritize rapid verification.

### 6.2. Comparison of Safety and Functionality

Table 3 below summarizes a comparison of the systems in terms of resistance to quantum attacks, linkability, anonymity, non-repudiation, support for off-chain payments, and applicability to blockchain.

(1) Quantum attack resistance

The proposed scheme relies on hash-based cryptographic primitives (e.g., Merkle trees and one-time signatures), making it inherently resistant to quantum computing attacks. This approach is similar to those in [25] (STARK scheme) and [26] (Gerky scheme), but provides stronger quantum resistance compared to [17] (which is not quantum-resistant).

(2) Linkability and anonymity

Our scheme achieves k-anonymity through a hybrid approach combining ring signatures and zero-knowledge proofs, ensuring unlinkability between transactions while permitting authorized tracing (e.g., for regulatory compliance). In contrast, some existing schemes (e.g., [18,27]) lack these properties, making it difficult to meet privacy protection requirements.

(3) Non-defamation

The proposed system enforces non-repudiation through digital signatures with proof-of-ownership, ensuring that a signer cannot deny authorship without compromising anonymity—similar to [24,26]. In contrast, [17,18,27] lack this property, which could prevent signers from effectively proving their innocence in certain scenarios.

(4) Support for off-chain payments

Designed explicitly for off-chain micropayments, our scheme employs optimized Hash Time-Locked Contracts (HTLCs) with low on-chain settlement overhead. Unlike most existing schemes (e.g., [24,25,26]), which do not support off-chain payments, only [17,18] offer partial applicability in this regard. However, reference [18] lacks anonymity and non-repudiation guarantees, while [17] is vulnerable to quantum attacks—introducing significant security limitations in both cases.

(5) Blockchain applicability

Our scheme is specifically designed for blockchain environments, similar to most related works ([17,18,24,27]), but offers distinct advantages over [25,26], which are incompatible with blockchain systems.

The proposed method demonstrates comprehensive superiority over existing solutions through three key advantages: (1) quantum-resistant security, (2) improved computational efficiency with reduced verification overhead, and (3) enhanced privacy protection featuring superior linkability control, non-repudiation guarantees, and off-chain payment support.

While requiring marginally longer signing times than some alternatives, this trade-off is justified by its exceptional verification efficiency—a critical requirement for blockchain applications. These characteristics make the scheme particularly suitable for large-scale deployment in privacy-sensitive scenarios, especially for off-chain payment systems demanding both high anonymity and operational performance.

### 6.3. Research Questions Answered

To validate the effectiveness of the proposed scheme, this section revisits the research questions raised in the introduction and explains how the experimental results support the claims.

RQ1: How do we construct a post-quantum secure, linkable hash-based ring signature scheme for off-chain IoT payments?

The proposed scheme is built upon XMSS, a hash-based post-quantum digital signature standardized by NIST, which inherently provides strong quantum resistance. By integrating linkability into the ring signature structure and optimizing it for limited-resource IoT devices, the scheme achieves both post-quantum security and lightweight operability. Table 3 confirms that the scheme provides full support for off-chain payments and blockchain applicability while maintaining linkability and anonymity, making it suitable for IoT scenarios.

RQ2: How do we efficiently detect double-spending while preserving user anonymity?

The proposed scheme introduces a linkability mechanism that allows identifying whether two signatures come from the same signer, without revealing the signer’s identity. As demonstrated in Table 3, this ensures both anonymity and linkability, enabling effective detection of double-spending attacks. Furthermore, the extremely low verification time (0.000947 s) supports fast and scalable checking of payment records.

RQ3: What are the performance and security advantages of our scheme compared to existing LRS solutions?

The comparison in Table 2 and Table 3 shows that, although the signature time (1.97 s) is higher than in some schemes, the verification time is significantly lower, which is critical for real-time payment confirmation in blockchain systems. Moreover, unlike many existing schemes, the proposed method simultaneously provides post-quantum security, linkability, anonymity, and non-repudiation, forming a complete and robust privacy-preserving solution for off-chain payment systems.

In summary, the experimental results and comparative analysis demonstrate that the proposed scheme successfully addresses the initial research questions in terms of security, performance, and applicability to real-world blockchain–IoT scenarios.

## 7. Conclusions

This paper proposes a post-quantum linkable ring signature scheme based on hash functions, specifically designed for off-chain payment scenarios in blockchain environments. By integrating post-quantum signature technology with linkable ring signatures, the scheme not only resists quantum computing threats but also effectively ensures the anonymity and traceability of payment transactions. Furthermore, through the development of a variant of Hash Time-Locked Contracts (HTLCs) that incorporates the latest State of Knowledge (SoK), the privacy of payment channels is further enhanced, reducing the risk of privacy leakage in practical off-chain payment applications.

### 7.1. Limitations

The limitations of this paper include, first, that the current scheme relies on hash-based post-quantum cryptography, and the computational overhead of its signature/verification process may be higher than that of classical signature schemes; second, this paper has consensus mechanism dependence. Similar to most blockchain applications, the system performance may be affected by the underlying consensus mechanism (such as Bitcoin, Ethereum, etc.), especially in terms of transaction final confirmation time and throughput.

### 7.2. Future Work

In terms of future research work, efforts can be directed towards the following aspects:

(1) Efficiency optimization: Developing lightweight signature schemes to improve the scalability of high-frequency payment networks.

(2) Cross-chain compatibility: Investigating interoperability with heterogeneous blockchain networks (such as hyperledger-permissioned chains and public chains).

(3) Enhanced privacy integration: Exploring the synergistic effects with secure multi-party computation (MPC) or fully homomorphic encryption (FHE) to construct a multi-layer privacy protection system that goes beyond the current linkable ring signature framework.

Security and performance analysis indicate that the scheme achieves a balance between privacy and efficiency while resisting quantum threats. These research results lay the foundation for a new generation of off-chain private payment systems.

## Figures and Tables

**Table 1:** XMSS parameters.

Introduction of Parameters
*h*: height of Merkle tree
*M*: hash digest value of the message
H: hash function
(sk,pk): pair of keys
${\mathrm{node}}_{i}$: node *i* of Merkle tree
Root: central node of the Merkle tree
σ: connectable ring signatures
$\left(x_{i},y_{i}\right)$: one-time signed key pairs
$\sigma_{\mathrm{OTS}}$: one-time signatures
${\mathrm{auth}}_{i}$: authentication path for signature creation by node *i*

**Table 2:** Comparison of program signature efficiency.

Scheme	Description	Members	Key Generation Time (s)	Signing Time (s)	Verification Time (s)
OURS	Based on hash functions	$2^{10}$	2.06	1.97	$9.47\times 10^{-4}$
[24]	Based on STARK	$2^{10}$	—	0.16	0.112
[18]	Based on hash functions	—	—	0.06	0.06
[26]	Geographically conditioned	$2^{10}$	—	4.864	3.584

**Table 3:** Comparison of signature scheme attributes.

Scheme	Quantum Resistance	Linkability	Anonymity	Non-Defensible	Off-Chain Processing	Blockchain
OURS	✓	✓	✓	✓	✓	✓
[24]	✓	✓	✓	✓	✗	✓
[27]	✓	✗	✗	✗	✗	✓
[17]	✗	✗	✗	✗	✓	✓
[25]	✓	✓	✓	✓	✗	✗
[18]	✓	✗	✗	✗	✓	✓
[26]	✓	✓	✓	✓	✗	✗

## Data Availability

Data are contained within the article.
